# Clostridial C3 Toxins Enter and Intoxicate Human Dendritic Cells

**DOI:** 10.3390/toxins12090563

**Published:** 2020-09-01

**Authors:** Maximilian Fellermann, Christina Huchler, Lea Fechter, Tobias Kolb, Fanny Wondany, Daniel Mayer, Jens Michaelis, Steffen Stenger, Kevin Mellert, Peter Möller, Thomas F. E. Barth, Stephan Fischer, Holger Barth

**Affiliations:** 1 Institute of Pharmacology and Toxicology, University of Ulm Medical Center, 89081 Ulm, Germany; maximilian.fellermann@uni-ulm.de (M.F.); christina.huchler@t-online.de (C.H.); lea.fechter@googlemail.com (L.F.); tobias-1.kolb@uni-ulm.de (T.K.); 2 Institute of Biophysics, Ulm University, 89081 Ulm, Germany; fanny.weiss@uni-ulm.de (F.W.); jens.michaelis@uni-ulm.de (J.M.); 3 Institute for Medical Microbiology and Hygiene, University of Ulm Medical Center, 89081 Ulm, Germany; daniel.mayer@uniklinik-ulm.de (D.M.); steffen.stenger@uniklinik-ulm.de (S.S.); 4 Institute of Pathology, University of Ulm Medical Center, 89081 Ulm, Germany; kevin.mellert@uni-ulm.de (K.M.); peter.moeller@uniklinik-ulm.de (P.M.); thomas.barth@uniklinik-ulm.de (T.F.E.B.)

**Keywords:** dendritic cells, clostridial C3 toxins, cellular uptake, C3bot_E174Q_, fusion toxin C2IN-C3lim, stimulated emission depletion (STED), super-resolution microscopy

## Abstract

C3 protein toxins produced by *Clostridium (C.) botulinum* and *C. limosum* are mono-ADP-ribosyltransferases, which specifically modify the GTPases Rho A/B/C in the cytosol of monocytic cells, thereby inhibiting Rho-mediated signal transduction in monocytes, macrophages, and osteoclasts. C3 toxins are selectively taken up into the cytosol of monocytic cells by endocytosis and translocate from acidic endosomes into the cytosol. The C3-catalyzed ADP-ribosylation of Rho proteins inhibits essential functions of these immune cells, such as migration and phagocytosis. Here, we demonstrate that C3 toxins enter and intoxicate dendritic cells in a time- and concentration-dependent manner. Both immature and mature human dendritic cells efficiently internalize C3 exoenzymes. These findings could also be extended to the chimeric fusion toxin C2IN-C3lim. Moreover, stimulated emission depletion (STED) microscopy revealed the localization of the internalized C3 protein in endosomes and emphasized its potential use as a carrier to deliver foreign proteins into dendritic cells. In contrast, the enzyme C2I from the binary *C. botulinum* C2 toxin was not taken up into dendritic cells, indicating the specific uptake of C3 toxins. Taken together, we identified human dendritic cells as novel target cells for clostridial C3 toxins and demonstrated the specific uptake of these toxins via endosomal vesicles.

## 1. Introduction

Bacterial C3 protein toxins specifically mono-ADP-ribosylate the small Rho-GTPases Rho A, B, and C at the amino acid residue Asn-41 [1]. In total, eight C3 isoforms produced from different bacterial strains have been identified [2], which possess similar molecular masses of about 25 kDa [3]. Moreover, the glutamic acid residue at position 174 (without signal sequence), which is important for the ADP-ribosylation activity, is conserved in the C3 ADP-ribosyltransferase family [1]. C3 produced by *Clostridium (C.) botulinum* (C3bot) was first described and thereby is the prototype of the whole C3-like ADP-ribosyltransferase family [4]. Later, a second C3 toxin was purified from *C. limosum* (C3lim) [5]. Despite obvious similarities of the C3 family members to A-subunits of AB-type toxins, no corresponding B-subunits have been identified for the C3 proteins so far [1,6]. Some exceptions are C3-like toxins such as PlxA and C3larvin, where a B subunit is suspected to be responsible for specific uptake into cells [7,8,9]. In consequence, classical C3 toxins are poorly internalized into most cell types, requiring high C3 concentrations (400–4000 nM) and long incubation times (>24 h) [5,10]. We and others found earlier that this is not the case for monocytes or macrophages, which show a tenfold increase in C3bot-sensitivity compared to fibroblasts or epithelial cells [11,12]. C3 toxins are internalized most likely into the endosomes of these cells and released into their cytosol, which is probably triggered by the acidification of maturating endosomes [11]. Furthermore, it has recently been shown that intermediate filament protein vimentin may also play a role in the uptake of C3 toxins in target cells [13,14]. However, the exact mechanism is not clarified yet and it is not known whether vimentin acts as a receptor to trigger endosomal uptake. In the cytosol, the C3 toxins ADP-ribosylate Rho, which disables Rho-signaling and thereby leads to several drastic consequences like the inhibition of cell-cycle progression [15], blocks exo- or endocytosis [6,16,17] and causes morphological changes by interaction with the actin cytoskeleton [18,19]. Due to their substrate specificity, C3 toxins are excellent tools to investigate or pharmacologically modify Rho signaling pathways [1]. To overcome the poor uptake of C3 toxins into most cell types, different approaches like microinjection [20,21], coupling to cell penetrating peptides [22,23,24], or generation of fusion toxins [19,25] have been used. One recombinant C3-based fusion toxin was generated as a chimera with the C3bot N-terminally fused to the binding and transmembrane domains of the diphtheria toxin (DT) [25]. However, here the use is restricted to cells expressing the diphtheria toxin receptor. For another fusion toxin (C2IN-C3lim), C3lim was C-terminally fused to C2IN, which is the enzymatically inactive N-terminal part of the *C. botulinum* C2 toxin’s A-subunit (C2I). In combination with the activated binding and translocation (B)-subunit (C2IIa), C2IN-C3lim can efficiently reach the cytosol of target cells via the C2 toxin transport mechanism [19]. For this second approach, a much broader range of cell types can be addressed due to the ubiquitous presence of the C2-toxin receptor [1,26]. Furthermore, our group showed that C2IN-C3lim alone is more efficiently taken up into the cytosol of monocytes, macrophages, or osteoclasts compared to C3bot or C3lim [6,27,28]. The uptake of active C3 toxins (including recombinant C2IN-C3lim) into monocytes or macrophages leads to alterations of the actin cytoskeleton, which causes characteristic morphological changes to these cells [11]. The pathophysiological role of C3 toxins is largely unknown. It is imaginable that C3 toxins negatively affect specialized cells of the immune system to lever out host defense. C3 treatment leads among others to reduced cytotoxicity of immune cells [22] and inhibits the migration and phagocytosis properties of macrophages and monocytes [11,29]. These effects can be exploited to target the pharmacological inhibition of the enhanced recruitment of monocytes/macrophages into damaged tissue in vivo, e.g., the alveolar space after blunt chest trauma [12,27], in order to reduce the local and systemic inflammation.

Here, we demonstrate that human dendritic cells (DCs) respond to clostridial C3 toxins ex vivo and specifically internalize these toxins into early endosomes. Therefore, the effects of different C3 toxins were investigated on a human dendritic cell sarcoma (U-DCS) cell line, which was developed, characterized, and established in Ulm and on human primary monocyte-derived DCs. Generally, DCs are involved in many immunological processes and diseases, since the migration of DCs into lymph nodes is an important prerequisite for antigen presentation and priming T-cells [30,31]. Therefore, DCs interconnect the innate and the adaptive immune system [32]. Our results not only extend the current knowledge about the pathophysiological role of clostridial C3 toxins as immune cell modulating toxins but also pave the way for potentially novel pharmacological strategies. Recombinant C3 toxins might serve for the targeted pharmacological modulation of Rho-dependent immune responses. The use of modified bacterial protein toxins for therapeutic purposes was already successfully demonstrated for other toxins such as botulinum neurotoxins (BoNTs) [33] and DT. For DT, the non-toxic mutant cross reactive material 197 (CRM197) is extensively used as a vaccine carrier [34,35] and a chimeric DT fusion protein (DAB_389_IL2, ONTAK^®^) is an immunotoxin and a licensed drug for the therapy of chronic lymphocytic leukemia [36,37,38].

## 2. Results

### 2.1. Selective Internalization of Clostridial C3 Toxins into the Cytosol of an Immortalized Human U-DCS Cell Line

**Figure 1:**
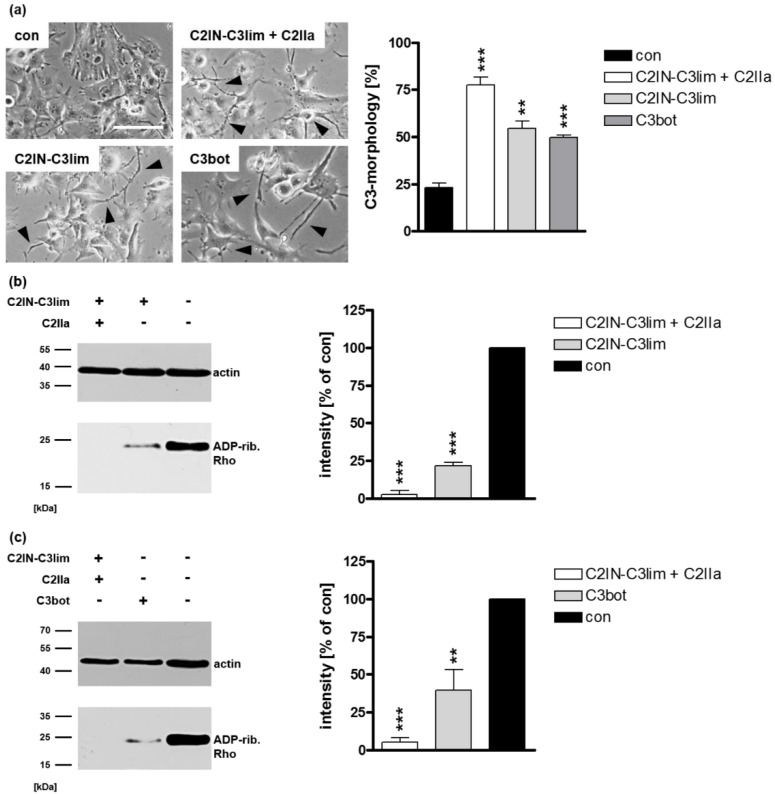
Effect of C3 toxins on human U-DCS cell line (**a**) U-DCS cells were treated with C2IN-C3lim (80 nM), C3bot (200 nM) or were left untreated (con). For a positive control, U-DCS cells were treated with the combination of C2IN-C3lim/C2IIa (40/66 nM). Representative phase contrast images after 5 h are shown. Scale bar corresponds to 50 µm and holds for all images. For quantitative analysis, the percentage of U-DCS cells displaying C3 morphology (marked with black arrow heads) was determined. Values are given as mean ± standard deviation (SD) (*n* = 3). (**b**) Cultured U-DCS cells were treated with C2IN-C3lim/C2IIa (40/66 nM), C2IN-C3lim (80 nm) or were left untreated (con) at 37 °C. (**c**) Cultured U-DCS cells were treated with C2IN-C3lim/C2IIa (40/66 nM), C3bot (200 nM) or were left untreated (con) at 37 °C. (**b**,**c**) After 5 h incubation time, cells were washed, lysed, and incubated with the respective freshly added C3 (300 ng) and biotin-NAD^+^ (10 µM) for 30 min at 37 °C. Afterwards, lysates were transferred to SDS-PAGE and Western blot analysis was performed to visualize biotin-labeled, i.e., ADP-ribosylated Rho proteins (~21 kDa), via peroxidase-coupled streptavidin (lower panels). Equal protein loading was confirmed via actin-immunostaining (upper panels, ~40 kDa). Densitometrical analyses from several experiments (normalized to actin loading control) are given as mean ± SD (*n* = 5). Significance was tested using a Student’s *t* test (** *p* < 0.01, *** *p* < 0.001).

By using an immortalized human U-DCS cell line, the effects of the chimeric fusion toxin C2IN-C3lim and C3bot on these cells were investigated. To examine the uptake, U-DCS cells were treated with C2IN-C3lim or C3bot. The changes in cell morphology due to actin rearrangement after treatment with C3 toxin is a specific readout to monitor the successful uptake of C3 toxins into the cytosol of target cells [6,11,18,39]. As a control, U-DCS cells were incubated with the combination of C2IN-C3lim plus C2IIa or were left untreated. Of note, the combination of C2IIa + C2IN-C3lim is not cell-type selective and is therefore taken up into all cells carrying C2 toxin receptors [19]. As shown in Figure 1a, U-DCS cells treated with C2IN-C3lim plus C2IIa showed the characteristic C3 toxin-induced changes in cell morphology. C3-induced inactivation of intracellular Rho results in characteristic changes in cell morphology based on the reorganization of the actin cytoskeleton. These changes are a well-established and sensitive endpoint to monitor the uptake of C3 toxins [11,19]. Remarkably, these changes were also visible when the cells were treated with C2IN-C3lim and C3bot alone.

To confirm this result, biochemical analyses were performed. If the C3 toxins were internalized efficiently into the cytosol of U-DCS cells, almost all intracellular Rho is ADP-ribosylated in the living cells. Therefore, modified Rho is no longer susceptible for subsequent in vitro ADP-ribosylation with biotin-labelled NAD^+^ as a co-substrate resulting in a weak signal in the Western blot analyses. For C2IN-C3lim (Figure 1b) as well as for C3bot (Figure 1c), a clear reduction of the signal intensity in the Western blot analyses was observable. Both the changes in cell morphology as well as the biochemical analyses of Rho ADP-ribosylation obviously indicate that the C3 toxins are internalized and are enzymatically active in the cytosol of the novel U-DCS cell line.

**Figure 2:**
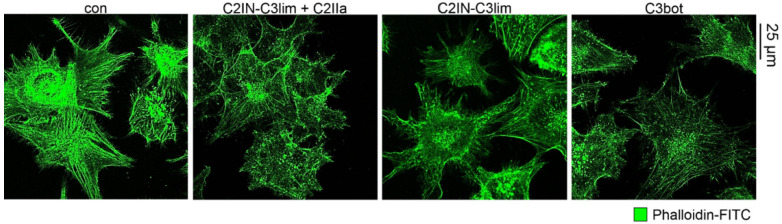
Treatment with C3 toxins affects the actin cytoskeleton of U-DCS cell line. Confocal microscopic analysis showing the intracellular reduction of F-actin bundles based on C3-induced ADP-ribosylation of Rho-proteins. U-DCS cells were incubated with C2IN-C3lim/C2IIa (80/132 nM), C2IN-C3lim (80 nM), C3bot (160 nM) or were left untreated (con). After 8 h, cells were washed, fixed, and permeabilized. F-actin was visualized using phalloidin-FITC. Scale bar corresponds to 25 µm and holds for all images.

As mentioned, Rho GTPases regulate the actin cytoskeleton [40]. Hence, we investigated whether the observed morphological changes are caused by a reorganization of filamentous actin (F-actin). Confocal microscopic images of phalloidin-FITC stained F-actin showed that actin bundles are clearly reduced in U-DCS cells treated with C2IN-C3lim or with C3bot compared to control cells (Figure 2). Moreover, C3 treatment seemed to reduce the overall signal intensity. These structural alterations confirm our previous results indicating the cellular internalization of C3 toxins into the cytosol of U-DCS cells.

**Figure 3:**
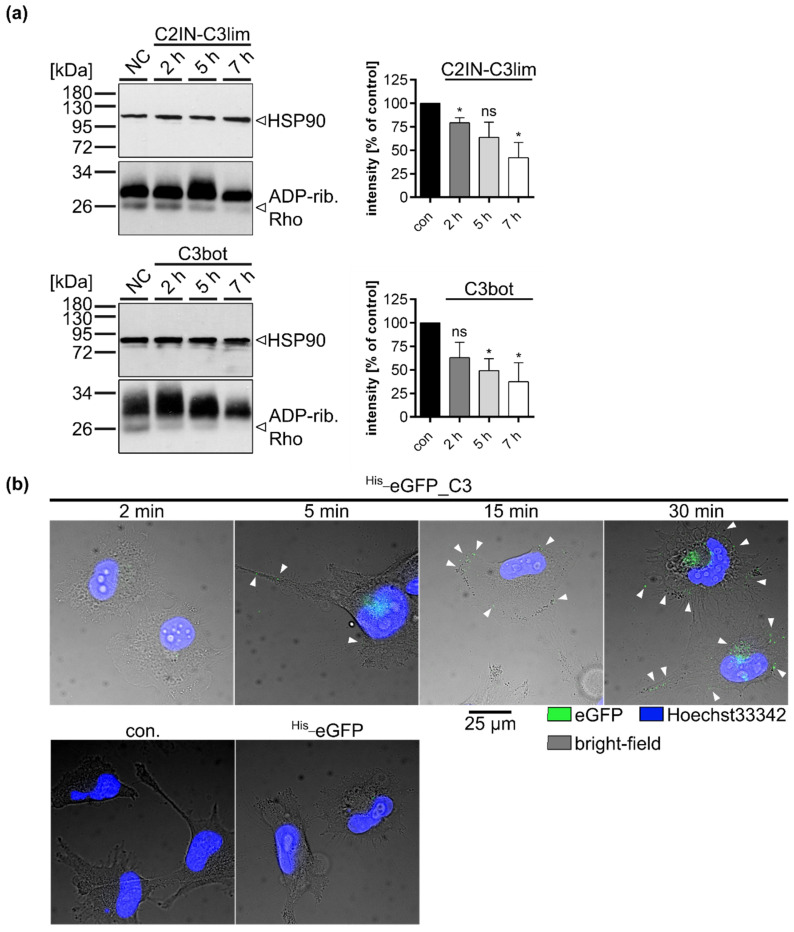
C3 toxins enter U-DCS cells in a time-dependent manner. (**a**) U-DCS cells were treated with C2IN-C3lim (80 nM), C3bot (200 nM) or were left untreated (con) at 37 °C. After 2, 5, or 7 h incubation time, cells were washed twice with PBS, lysed, and incubated with freshly added C3bot (15 pmol) and biotin-NAD^+^ (10 µM) for 30 min at 37 °C. Afterwards, lysates were transferred to SDS-PAGE and Western blot analyses to visualize biotin-labeled, i.e., ADP-ribosylated Rho proteins via peroxidase-coupled streptavidin (lower panels, ~21 kDa). Equal protein loading was confirmed via HSP90-immunostaining (upper panels, ~90 kDa). Densitometrical analyses from several experiments (normalized to HSP90 loading control) are given as mean ± SD (*n* = 3). Significance was tested using a Student’s *t* test (ns = not significant, * *p* < 0.05). (**b**) U-DCS cells were treated with ^His_^eGFP_C3bot (250 nM) for 2, 5, 15, 30 min, for 30 min with ^His_^eGFP, or were left untreated (con). Subsequently, the cells were washed twice with PBS and fixed with PFA. After nucleus staining with Hoechst33342 (blue) images were taken with an epifluorescence microscope. The three channels for Heechst33342 at 390 nm excitation blue, for eGFP at 488 nm excitation, and the bright-field (for cell borders) were merged. Scale bar corresponds to 25 µm and holds for all images. For better visualization, green eGFP-signals were marked with white arrow heads.

Next, we wanted to examine time-dependency of the uptake of C3 toxins into the U-DCS cells. U-DCS cells were treated with either C2IN-C3lim (Figure 3a, upper panel) or with C3bot (Figure 3a, lower panel) and the ADP-ribosylation status of Rho was checked by Western blot analyses after different time points. A clear decrease was observed for the signal of post-ADP-ribosylated Rho indicating a time-dependent uptake and cytosolic Rho ADP-ribosylation in intact cells. To further visualize the time-dependent uptake process, a green fluorescent protein (eGFP) was N-terminally fused to C3bot (^His_^eGFP_C3bot). After just five minutes, first punctual, probably vesicular signals on the cell membrane became visible. (Figure 3b). The number of these signals increased over time. It is noteworthy that for the fluorescent-labeled eGFP alone, only negligible amounts of signals could be detected even after 30 min, excluding label-mediated uptake in U-DCS cells.

The U-DCS cell line was used to evaluate the cytosolic uptake of C3 toxins into DCs. Next, we wanted to confirm the observations in a different model. Therefore, ex vivo isolated human monocyte-derived DCs were generated and their C3 uptake properties were studied.

### 2.2. Clostridial C3 Toxins Are Internalized into the Cytosol of Immature and Mature Human Monocyte-Derived DCs

Primary human monocytes can be differentiated to immature and mature DCs by treatment with cytokines and hormone-like molecules (see Experimental procedures). First, immature DCs were incubated with C2IN-C3lim (80 nM) and as control, with C2IN-C3lim/C2IIa (5/8.5 nM). After 3.5 h, a clear change in cell morphology was visible indicating a successful internalization of the fusion toxin into the cytosol of immature DCs (see Supplementary Figure S1a). These results were confirmed via Western blot analysis of Rho ADP-ribosylation (see Supplementary Figure S1b). Moreover, the findings could be extended to C3bot (160 nM) as well as C3lim (160 nM), (see Supplementary Figure S1c).

**Figure 4:**
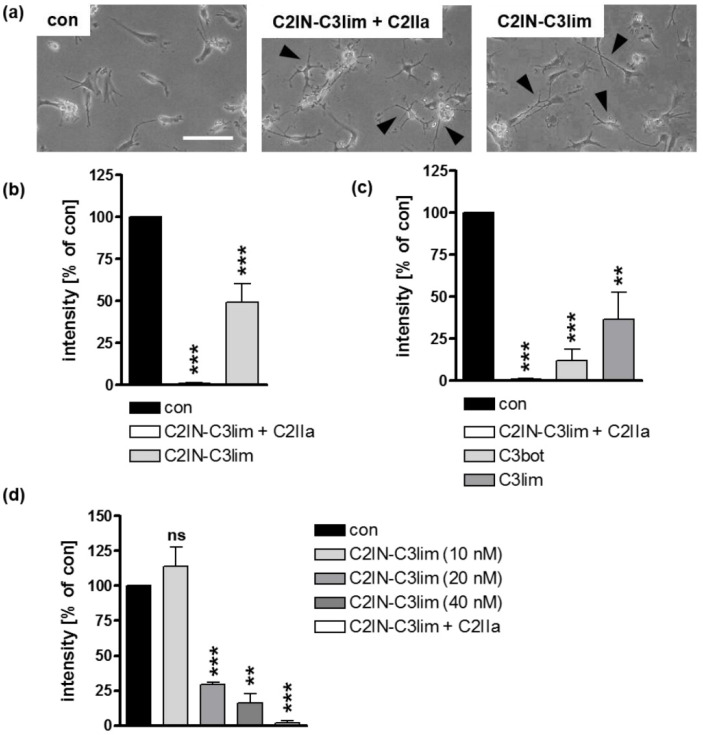
C3 toxins are internalized into the cytosol of mature human monocyte derived DCs. (**a**) Mature human DCs were treated at 37 °C with C2IN-C3lim/C2IIa (5/8.5 nM), C2IN-C3lim (80 nM), or were left untreated (con). Representative phase contrast images after 3.5 h are shown. Scale bar correspond to 50 µm and holds for all images. Cells displaying obvious C3-morphology are marked with black arrow heads. (**b**) Subsequently, cells were washed, lysed, and incubated with freshly added C3 (300 ng) and biotin-NAD^+^ (10 µM) for 30 min at 37 °C. Afterwards, cell lysates were transferred to SDS-PAGE and Western blot analysis to detect biotin-labeled, i.e., ADP-ribosylated Rho proteins via peroxidase-coupled streptavidin. Equal protein loading was confirmed via Ponceau S staining of the membrane. Densitometrical analyses from several experiments (normalized to Ponceau S loading control) are given as mean ± SD (*n* = 4). (**c**) Mature DCs were treated at 37 °C with C2IN-C3lim/C2IIa (5/8.5 nM), C3bot (160 nM), C3lim (160 nM), or were left untreated (con). After 3.5 h, detection and densitometric quantification of ADP-ribosylated Rho was performed as described above (*n* = 4) (**d**) Mature DCs were treated at 37 °C with decreasing concentrations of C2IN-C3lim ranging from 40 nM over 20 nM to 10 nM. As a control, cells were treated either with C2IN-C3lim/C2IIa (10/17 nM) (positive control) or were left untreated (con). After 3 h, detection and densitometric quantification of ADP-ribosylated Rho was performed as described above (*n* = 2) (**b**–**d**) Significance was tested using a Student’s *t* test (ns = not significant, ** *p* < 0.01, *** *p* < 0.001).

Immature DCs engulf various proteins and peptides, while mature DCs lose the ability for non-specific pinocytosis [41,42]. Therefore, we investigated whether C3 toxins are also taken up into the cytosol of mature human DCs. Mature DCs were treated with C2IN-C3lim (80 nM), C2IN-C3lim/C2IIa (5/8.5 nM), or were left untreated. After 3.5 h, mature DCs showed distinct C3-induced morphological changes (Figure 4a) as well as ADP-ribosylation of Rho (Figure 4b). Likewise, C3bot (160 nM) or C3lim (160 nM) ADP-ribosylated Rho within 3.5 h (Figure 4c). As proof of concept, a concentration-dependent effect of Rho ADP-ribosylation was shown for C2IN-C3lim in mature DCs (Figure 4d). In conclusion, C3 toxins are rapidly internalized into the cytosol of immature and mature human DCs. However, the specificity of this molecular uptake has yet to be confirmed. Therefore, we investigated whether proteins or single A-subunits of other bacterial AB-type toxins in general enter the cytosol of DCs via an unspecific uptake mechanism. In order to clarify this issue, we used the enzymatically active component C2I of the *C. botulinum* C2 toxin and incubated U-DCS cells as well as monocyte-derived immature or mature DCs with C2I in the absence of C2IIa which will be described in detail in the next section.

### 2.3. Confirming the Selectivity and Specificity of the Uptake of C3 Toxins into DCs

**Figure 5:**
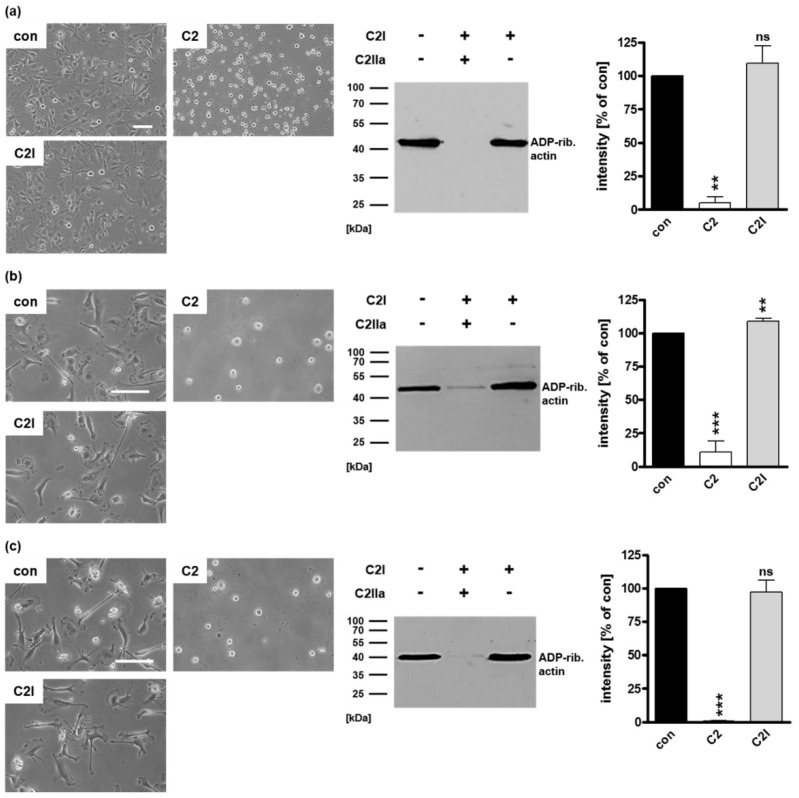
The enzyme component C2I alone does not enter DCs. (**a**) U-DCS cells were incubated with C2I (80 nM), with complete C2 toxin (40/66 nM), or left untreated (con). After 5 h, representative phase contrast images were taken, cells were washed, lysed, and mixed with biotin-NAD^+^ (10 µM) and fresh C2I (300 ng) for 30 min at 37 °C. Subsequently, biotinylated, i.e., ADP-ribosylated actin (~40 kDa) was detected using peroxidase-coupled streptavidin in Western blot analysis. Equal protein loading was confirmed via Ponceau S staining of the membrane. Densitometrical analyses from two experiments (normalized to Ponceau S loading control) are given as mean ± SD. Immature (**b**) as well as mature (**c**) human monocyte-derived DCs were incubated with C2I (20 nM) without C2IIa, with complete C2 toxin (2/3.3 nM) or left untreated (con). After 5 h, representative phase contrast pictures are shown. Then, the cells were washed, lysed, and incubated with biotin-NAD^+^ (10 µM) and fresh C2I (300 ng) for 30 min at 37 °C. Next, Western blot detection of biotinylated, i.e., ADP-ribosylated actin (~40 kDa) was performed. Densitometrical analyses from three experiments (normalized to Coomassie-stained SDS-gel used as loading control) are given as mean ± SD. (**a**–**c**) Scale bars correspond to 50 µm and hold for all images. Significance was tested using a Student’s *t* test (ns = not significant, ** *p* < 0.01, *** *p* < 0.001).

The enzyme subunit C2I of the binary *C. botulinum* C2 toxin is not able to enter cells in the absence of its separate transport subunit C2IIa. Therefore, C2I was used to investigate whether ADP-ribosylating enzymes such as C3 toxins are taken up by DCs just via a non-specific mechanism. U-DCS cells were incubated with complete C2 toxin (C2I + C2IIa, 40/66 nM) as a control or with C2I (80 nM) in the absence of C2IIa for 5 h (Figure 5a). The cytosolic uptake of C2I leads to the ADP-ribosylation of intracellular globular (G)-actin. This ADP-ribosylation results in the breakdown of the actin cytoskeleton which leads to drastic morphological changes, i.e., cell rounding [43,44,45]. Neither morphologically nor biochemically was an uptake of C2I into U-DCS cells observed in the absence of C2IIa (Figure 5a). However, control U-DCS cells treated with complete C2 toxin showed the expected cell rounding and ADP-ribosylation of actin (Figure 5a). The same set of experiments was applied for immature (Figure 5b) and mature (Figure 5c) DCs. The results revealed no detectable uptake of C2I into the cytosol of primary DCs regardless of their maturation status. Taken together, we exclude general unspecific uptake of ADP-ribosyltransferases via pinocytosis. In none of the cases investigated so far was the enzyme component C2I able to enter DCs. In contrast, for the several C3 toxins the uptake into the cytosol of DCs was demonstrated by different approaches. Furthermore, we show that uptake of C3 into the cytosol of cells is specific for cells of the monocytic lineage and does not take place in, for example, epithelial cells (see Supplementary Figure S2). However, the underlying molecular mechanism is not clear so far; therefore, we investigated, whether the C3 toxins enter DCs via intracellular trafficking across early endosomes.

### 2.4. Specific Internalization of C3bot into Early Endosomes of Mature and Immature DCs

**Figure 6:**
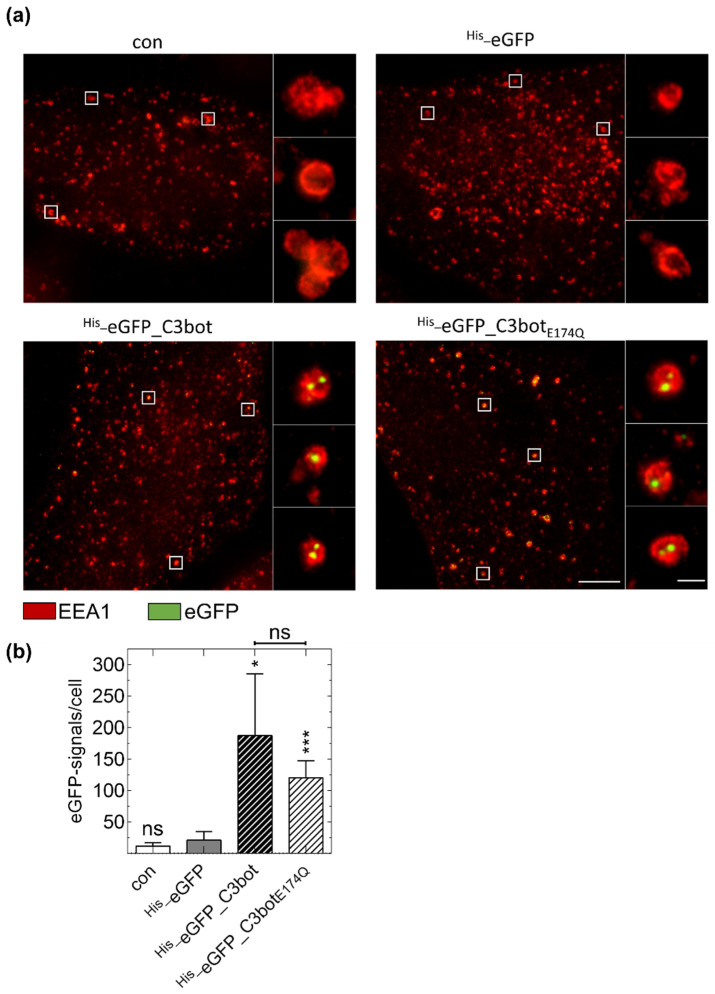
Specific internalization of eGFP (green)-labeled C3bot and C3bot_E174Q_ into early endosomes (red) of mature human monocyte-derived DCs ex vivo. (**a**) Isolated human monocytes were differentiated into mature DCs. DCs were treated with 250 nM ^His_^eGFP_C3bot, ^His_^eGFP_C3bot_E174Q_, ^His_^eGFP for 30 min at 37 °C, or left untreated (con). After subsequent immunostaining, STED super-resolution microscopic images were captured. Magnified areas of each image are marked with white squares. The experiment was repeated with DCs differentiated from monocytes of five individual and independent donors. Scale bar corresponds to 5 µm (0.5 µm for the magnifications) and holds for all images. (**b**) The detected green spots were quantified for each treatment (*n* = 5 donors). Significant differences compared to the ^His_^eGFP samples were tested using a Student’s *t* test (ns = not significant, * *p* < 0.05, *** *p* < 0.001). Comparing the samples with ^His_^eGFP_C3bot and ^His_^eGFP_C3bot_E174Q_ no significant differences were found as indicated above the graph.

Next, we investigated whether the molecular uptake of C3bot by human DCs resembles the uptake of C3 toxins by monocytes/macrophages, i.e., receptor-mediated endocytosis and release from acidified early endosomes. Additionally, we aimed to use C3bot and its enzymatically inactive, i.e., non-toxic mutant C3bot_E174Q_ as a monocyte-derived immune cell specific molecular transporter to selectively introduce cargo molecules into these cells. To address both points, eGFP-labelled C3bot (^His_^eGFP_C3bot) or C3bot_E174Q_ (^His_^eGFP_C3bot_E174Q_) were generated and the endosomal uptake was investigated. Using dual-color stimulated emission depletion (STED) super-resolution optical microscopy we show that both C3bot proteins are internalized into immature (Supplementary Figure S3) as well as mature DCs (Figure 6). By magnification of the STED super-resolution image (Figure 6, panels on the right of the main image), the C3 proteins were localized in the inner lumen of early endosomes as indicated by co-staining of the early endosomal antigen 1 (EEA1). Notably, when the DCs were treated with ^His_^eGFP alone the signals for internalized eGFP were strongly reduced and undetectable in the negative control. Hence, it is unlikely that the C3bot proteins are internalized in a non-specific process, since eGFP is only efficiently internalized when coupled to C3bot or C3bot_E174Q_. Moreover, uptake is also noted for the non-toxic ^His_^eGFP_C3bot_E174Q_ mutant strongly supporting the use of C3 proteins as promising cargo-transporters into DCs. In conclusion, C3 proteins are specifically internalized into early endosomes suggesting a similar uptake-mechanism as for monocytes/macrophages.

Taken together, the result demonstrates that non-toxic C3 proteins might serve as a transporter for the successful and specific delivery of “foreign” proteins and peptides such as eGFP into DCs.

## 3. Discussion

In the present study, we demonstrated that DCs apart from monocytes or macrophages are target cells of ADP-ribosylating C3 transferases. The U-DCS cell line as well as human ex vivo isolated monocyte-derived DCs showed concentration-dependent ADP-ribosylation of Rho GTPases when C3 toxins were added into the culture medium. Moreover, this inactivation of Rho induced a characteristic morphological change, i.e., the formation of long and thin cellular protrusions as well as a reduction of actin bundles similar to that described for monocytes and macrophages [11]. Furthermore, these results confirm earlier investigations of Rho-signaling in DCs, where similar morphological changes were observed [46,47,48]. However, two of these studies used microinjection to introduce C3 into DCs, probably because at this time it was believed that C3bot could not enter cells efficiently [5,10]. To the best of our knowledge, only one publication has investigated the ADP-ribosylation of Rho in DCs after the extracellular application of C3bot [47]. However, the uptake mechanism of C3bot was not investigated in detail. In addition, it was not examined whether C3 was taken up specifically or perhaps non-specifically via pinocytosis [47]. In the present study we show that C3 is internalized specifically into DCs, while a similar ADP-ribosyltransferase (C2I), and ^His_^eGFP are not. It must be mentioned that there is still a fundamental lack of knowledge about the uptake and intracellular trafficking of C3 toxins and further research is needed here especially in identifying the host cell receptor of C3 toxins. Once the receptor or the receptors are identified, further studies confirming the specific uptake must be performed. Indeed, the tested C3 toxins are not only internalized into immature but also into mature DCs, which probably have lost the ability for non-specific pinocytosis during maturation [41,42]. Additionally, the presented STED super resolution microscopic data suggest that C3bot is internalized into early endosomes, indicating a similar mechanism as that suggested for monocytes or macrophages, i.e., the release from acidified endosomes [11].

DCs play a crucial role in both the innate and the adaptive immune system. During DC maturation, antigens (Ags) are internalized, processed (e.g., cleavage of larger proteins), and presented to major histocompatibility complex (MHC) class I and MHC class II molecules [32,49]. In the classical pathways, MHC class I molecules present cytosolic Ags, while MHC class II molecules bind Ags processed within endosomes [32,50]. As potent Ag-presenting cells, mature DCs migrate to lymphoid tissues and initiate T- and B-cell immune responses [30,31,32,51]. In earlier studies, it was shown that Rho GTPases (especially Rho A) are involved in these processes, i.e., antigen presentation, podosome formation, and interaction with T-cells [47,48,49,52]. Our data suggest that C3 toxins are excellent tools to modulate DC-mediated immune responses. C3 is selectively internalized into DCs and specifically inhibits Rho proteins in the cytosol of these cells. Therefore, C3 toxins seem to be promising candidates for the pharmacological suppression of undesired DC-mediated immune responses, for example, in auto-immune diseases or transplant rejections [30,31,53]. In this context, C2IN-C3lim is the most promising candidate since it was shown to be more efficiently internalized compared to C3bot or C3lim. Furthermore, the C2IN-C3lim-mediated ADP-ribosylation is reversible due to de-novo synthesis of Rho, after degradation of cytosolic C2IN-C3lim [27,39]. Treatment reversibility is desired to restrict the time period and not completely lose DC-functions.

DCs play an important role in initiating an immune response and in fighting infectious diseases and are therefore a crucial target for pathogens [30,32,54]. Pathogenic bacteria that produce and secrete specific macrophage- or DC-inhibitory proteins might have an evolutionary advantage over others. We show that C3bot as well as its enzymatically inactive mutant C3bot_E174Q_ are specifically internalized into early endosomes of DCs. By coupling cargo proteins to C3bot_E174Q,_ the uptake of theses cargo molecules into DCs is enhanced, as shown for ^His_^eGFP. These internalized cargo proteins may then be processed in the endosomes or the cytosol, coupled to MHC class I or II molecules, and presented on the cell surface for activation of T- or B-cells. Hence, it seems plausible to investigate the use of C3bot_E174Q_ as a vaccine carrier or for the introduction of therapeutic (macro-) molecules specifically into DCs. Using enzymatically inactive toxin mutants as vaccine carrier is a common method and the most prominent example is the nontoxic mutant CRM197 of DT, which is well established and administered as carrier protein worldwide [34,35]. In this regard, we already demonstrated that different cargo molecules can infiltrate into the cytosol of macrophages or monocytes via C3bot_E174Q_-mediated transport [55,56,57]. Such an approach might be used to initiate immune responses against cancer cells as discussed for several types of cancers [30]. In conclusion, the C3 toxins C3bot and C2IN-C3lim as well as the enzymatically inactive mutant C3bot_E174Q_ are specifically internalized into DCs and should be promising candidates for the targeted pharmacological modulation and manipulation of immune cells.

## 4. Materials and Methods

### 4.1. Cell Culture

All cells were cultured at 37 °C, 5% CO_2_ and constant humidity. For U-DCS cell line, a medium mixed of IMDM (Lonza) and RPMI 1640 (Gibco-Life Technologies, Carlsbad, CA, USA) in a ratio of 4:1 was used and supplemented with 10% fetal calf serum (Gibco-Life Technologies, Carlsbad, CA, USA), 0.1% insulin-transferrin-sodium selenite supplement (Roche Diagnostics, Basel, SUI), 1% L-glutamine (Thermo Fisher Scientific, Waltham, MA, USA), and 100 U/mL (1%) penicillin–streptomycin (Gibco-Life Technologies, Carlsbad, CA, USA). Subconfluent cells were passaged after trypsinization (Roche Diagnostics, Basel, SUI) every 3 to 4 days and split in a ratio of 1:2 or 1:3.

### 4.2. Generation of Mature and Immature DCs Based on Isolated Human Monocytes

PBMC were isolated from buffy coats by Ficoll-Hypaque (BD Biosciences, Franklin Lakes, NJ, USA) density centrifugation. Plastic-adherent cells (monocytes) were stimulated with GM-CSF (20 ng/mL; Miltenyi, Bergisch Gladbach, Germany) and IL-4 (20 ng/mL; Biolegend, San Diego, CA, USA) in Macrophage-SFM (Gibco-Life Technologies, Carlsbad, CA, USA) for 7 to 8 days to obtain immature dendritic cells. To generate mature DCs monocytes were treated with GM-CSF and IL-4 as above, and IL-1β (2 ng/mL; R&D Systems, Minneapolis, MN; USA), Prostaglandin E2 (1 µg/mL; Sigma-Aldrich, St. Louis, MO, USA), IL-6 (10 ng/mL, R&D Systems, Minneapolis, MN, USA), and TNF-β (10 ng/mL, R&D Systems, Minneapolis, MN, USA) were added for an additional two days. The maturation status was confirmed using flow cytometry by detection of established cell surface markers [58]. The immature and mature DCs were harvested with 1 mM EDTA (Sigma-Aldrich St. Louis, MO, USA, Gibco-Life Technologies, Carlsbad, CA, USA) and resuspended in Macrophage-SFM.

### 4.3. Cloning of ^His_^eGFP-Labeled C3bot and C3bot_E174Q_

In the basic construct ^His_^eGFP was N-terminally fused to C3bot_E174Q_. Therefore, the DNA sequence for eGFP was amplified using a polymerase chain reaction and inserted into a plasmid backbone coding for C3bot_E174Q_ within the multiple cloning site. For the insertion, traditional cloning techniques depending on restriction enzymes (NcoI and BamHI) were used, while the restriction sites were hidden in the 6xHis-tag or the SGGGSGGGS-linker connecting eGFP and C3bot_E174Q_. Based on the resulting plasmid (pEG-His1: ^His_^eGFP_C3bot_E174Q_) the point mutation at position 174 (C3bot without signal sequence) was reverted using the QuikChange XL site-directed mutagenesis (Agilent Technologies, Santa Clara, CA, USA). The resulting construct (pEG-His1: ^His_^eGFP_C3bot) was used for the expression of ^His_^eGFP_C3bot. The sequence correctness of all cloned constructs was verified using GATC Sanger sequencing services (Eurofins, Luxembourg).

### 4.4. Protein Expression and Cell Lysis

Plasmids were transformed into competent *Escherichia coli* BL21. The first preculture with 5 mL LB-medium (1% tryptone, 0.5% yeast extract, 1% NaCl, 100 µg/mL ampicillin) was inoculated with one single colony and incubated at 37 °C and 180 rpm in a shaking incubator. A second overnight culture with 150 mL fresh LB-medium in an Erlenmeyer flask was inoculated with the total volume of preculture. The main culture of 4 × 1 L LB-medium in Erlenmeyer flasks were inoculated with the overnight preculture and incubated at 37 °C and 180 rpm until an OD_600_ of 0.6–0.8 was reached. Subsequently, target protein expression was induced by adding 0.5 mM Isopropyl β-d-1-thiogalactopyranoside (IPTG). The induced main culture was incubated at 16 °C (^His_^eGFP-labeled proteins) or 29 °C (GST-tagged proteins), 180 rpm over-night. Cells were harvested by centrifugation at 5500 rcf and 4 °C for 10 min. The pellet was resuspended in ether 40 mL lysis buffer (10 mM NaCl, 20 mM Tris, 1% Triton X-100, 1% phenylmethylsulfonyl fluoride (PMSF), pH 7.4) for the GST-tagged proteins or 40 mL NPI-20 (50 mM NaH_2_PO_4_, 300 mM NaCl, 20 mM imidazole, 1% PMSF, pH 8.0) for the His-tagged proteins. Subsequently, the cells were lysed by sonication (10 × 20 s pulses and intermediate pauses of 30 s). Not lysed cells and non-soluble membrane parts were removed by centrifugation at 13,000 rcf and 4 °C for 30 min and filtration of the supernatant through 0.45 µm and 0.2 µm syringe filters.

### 4.5. Purification of GST-Tagged Proteins

C3bot, C3lim, and C2IN-C3lim were purified as GST-tagged proteins like described earlier [19,27]. After overexpression, the filtered cell lysates were incubated with 1.2 mL in PBS (137 mM NaCl, 2.7 mM KCl 8 mM Na_2_HPO_4_ and 1.8 mM KH_2_PO_4_, pH 7.4) equilibrated Protino™ Glutathione Agarose 4B-beads (Macherey-Nagel™, Düren, Germany) for 1–2 h at room temperature (RT) or overnight at 4 °C. The beads were washed twice with washing buffer (150 mM NaCl, 20 mM Tris–HCl, pH 7,4) and once with PBS (centrifugation at 3000 rcf for 5 min). Afterwards, the proteins were eluted by cleaving off the GST-tag with thrombin (80 NIH units, produced by Amersham Biosciences, Little Chalfont, UK) for 1 h at RT. The glutathione-beads were removed by centrifugation at 10,000 rcf and 4 °C for 30 s. The remaining thrombin was removed by incubating the supernatant with 120 μL Benzamidine-Sepharose^®^ 6B-beads (GE Healthcare, Chicago, IL, USA) for 10 min at RT. The benzamidine-beads were removed by centrifugation at 10,000 rcf, 4 °C for 30 s and the concentration of the purified in PBS dissolved proteins was determined in SDS-PAGE in comparison to a BSA standard.

### 4.6. Purification of 6xHis-Tagged Proteins

The obtained soluble cell lysate was incubated with PureCube 100 INDIGO Ni-agarose (Cube Biotech, Monheim am Rhein, Germany) overnight at 4 °C. Using NPI-20 preequilibrated PureCube 1-step batch Midi Plus Columns (Cube Biotech, Monheim am Rhein, Germany) the Ni-agarose beads were collected and washed tree times with 20 mL NPI-20. The His-tagged proteins were eluted according to the PureCube 1-step batch Midi Plus Column protocol using NPI-250 (50 mM NaH_2_PO_4_, 300 mM NaCl, 250 mM imidazole, pH 8.0). After analysis of the different fractions in SDS-PAGE, the fractions with highest target protein content and lowest impurities were collected. The buffer containing imidazole was exchanged with PBS by at least three rounds of ultrafiltration (Vivaspin 20 centrifugal concentrator with 10 kDa molecular weight cutoff, produced by Merk KGaA, Darmstadt, Germany). The protein solutions were stored at −80 °C and the concentrations were determined in SDS-PAGE compared to a BSA standard.

### 4.7. Phase Contrast Microscopy

The cells were seeded in microtiter plates and incubated with the respective toxin for the indicated time at 37 °C. Three phase contrast images were taken for each treatment in separate wells and/or different areas within the well. Representative images are shown with scale bars.

### 4.8. SDS-PAGE and Western Blotting

The proteins were separated depending on their molecular weight in SDS-PAGE using 12.5% acrylamide gels. The proteins were transferred on a nitrocellulose membrane using semi-dry blot chamber. Subsequent to Ponceau S (AppliChem GmbH, Darmstadt, Germany) staining, unspecific binding was blocked by incubation in 5% skim milk powder diluted in PBS-T (137 mM NaCl, 2.7 mM KCl 8 mM Na_2_HPO_4_, 1.8 mM KH_2_PO_4_, 0.1% Tween^®^20, pH 7.4) for 1 h at RT. The membrane was quickly washed with PBS-T and incubated with actin monoclonal antibody (1:10,000, ACTN05(C4); Thermo Fisher Scientific, Waltham, MA, USA) or streptavidin-peroxidase conjugate (1:5000; Sigma-Aldrich, St. Louis, MO, USA) diluted in PBS-T for 1 h at RT. Afterwards, unbound proteins were removed by washing three times with PBS-T for 5 min at RT on an orbital shaker. For the detection of actin, the membrane was further incubated with the secondary goat anti-mouse antibody-HRP (1:2500; Santa Cruz Biotechnology, Dallas, TX, USA) for 1 h at RT and washed three times with PBS-T. The peroxidase marked proteins were detected using Pierce™ ECL Western Blotting Substrate (Thermo Fisher Scientific, Waltham, MA, USA) and X-ray films (AGFA Health Care, Mortsel, Belgium).

### 4.9. Sequential ADP-Ribosylation of Rho

The cells were seeded in microtiter plates and treated with C3 or C2 toxins for the indicated concentration and time. Subsequently, the cells were washed with PBS to remove the extracellular toxin. The cells were lysed in ADP-ribosylation buffer (20 mM Tris-HCl, 1 mM EDTA, 1 mM DTT, 5 mM MgCl_2_, cOmplete™ (1:50, freshly added), pH 7.5) by freezing and thawing on ice. Fresh C3 or C2 toxin (300 ng) and 6-biotin-17-NAD^+^ (10 μM) were added in excess and the reactions at 37 °C were started and stopped at the same time. The reactions were stopped after 30 min by the addition of a Laemmli buffer (0.3 M Tris-HCl, 10% SDS, 37.5% glycerol, 0.4 mM bromophenol blue) and heat denaturation. The samples were subjected to SDS-PAGE and Western blotting. Biotinylated, i.e., in cell lysate ADP-ribosylated Rho or actin was detected with streptavidin-peroxidase conjugate (1:5000). For densitometric analysis of the detected protein bands, ImageJ software (v1.52i) was used. Importantly, a weak signal indicates that most of the substrate was already ADP-ribosylated during treatment of intact cells with the respective toxin.

### 4.10. Immunofluorescence Staining with Following Confocal and Epifluorescence Microscopy

U-DCS cells were seeded overnight in 8-well plates (ibidi GmbH, Gräfelfing, Germany) with a density of 3 × 10^4^ or 4 × 10^4^ cells per well in a total volume of 300 µL. The cells were treated with the indicated protein/toxin for the indicated time at 37 °C and 5% CO_2_. Subsequently, the cells were washed with PBS and fixed with 4% paraformaldehyde for 20 min at RT. After permeabilization using 0.4% Triton X-100, the cells were blocked with 5% skim milk powder in PBS-T and afterwards incubated with Phalloidin-FITC (1:100) for 1 h. For nucleus staining, non-permeabilized cells were incubated in PBS containing Hoechst33342 (5 µg/mL) for 10 min at RT. After the staining process, the samples were washed three times with PBS to remove unbound dye. Confocal images were obtained using an LSM 710 laser scanning confocal microscope system (Zeiss, Oberkochen, Germany) equipped with a 63× oil immersion objective. Epifluorescence images were taken with an iMIC microscope, a 40× oil immersion objective and Live Acquisition 2.6 software (TILL Photonics GmbH–an FEI Company, Hillsboro, OR, USA). Obtained images were afterwards processed with ImageJ software (v1.52c).

### 4.11. STED Super-Resolution Microscopy

10^5^ mature or immature DCs were seeded per well of an 8 well µ-slide with glass bottom (ibidi GmbH, Gräfelfing, Germany). The cells were treated with 250 nM of the indicated protein (^His_^eGFP, ^His_^eGFP_C3bot or ^His_^eGFP_C3bot_E174Q_) for 30 min at 37 °C. Subsequently, the cells were washed twice with cold PBS and fixed in 3.2% paraformaldehyde in PBS (32% PFA aqueous solution, electron microscopy sciences) for 20 min at RT. The remaining PFA was removed by washing the samples three times with cold PBS. The cells ware permeabilized and unspecific binding was blocked by incubation in blocking solution (3% (*w*/*v*) BSA and 0.3% (*v*/*v*) TritonX-100 dissolved in PBS) for 2 h at RT. The samples were incubated overnight at 4 °C with 1 µg/mL of the primary rabbit anti-EEA1 antibody (1 mg/mL from Thermo Scientific, Waltham, MA, USA) and 0.5 μg/mL of Atto594-conjugated GFP-booster nanobody (0.5 mg/mL from Chromotek, Planegg-Martinsried, Germany) dissolved in 1:10 diluted blocking solution. After three washing steps, the samples were incubated with 1 µg/mL of the secondary goat anti-rabbit antibody conjugated with Atto647N (1 mg/mL from Sigma-Aldrich St. Louis, MO, USA) dissolved in 1:10 diluted blocking solution. Before imaging, the unbound antibodies were removed with three washing steps in PBS, which was finally exchanged by 2,2′-thiodiethanol (97% solution in PBS, pH 7.5). Images were captured using a self-build dual-color 3D STED microscope [59]. Typically, an average power of 0.8 µW per excitation beam and 1.3 mW per depletion beam were used. The STED images possess a pixel size of 12.5 nm and were captured with a dwell time of 300 µs and approximately 150 counts as typical peak photon number. Images were analyzed in ImageJ (v1.52n) and a Gaussian blur σ = 1 and an intensity threshold of >20 counts was applied for better visualization. For automated quantification of the eGFP-signals a threshold of >35 was applied and a programed search algorithm in Python 3.7 was used. The mean number of signals per cells per donor was calculated and averaged for 5 donors (*n* = 5).

## Supplementary Materials

The following are available online at https://www.mdpi.com/2072-6651/12/9/563/s1. Figure S1: C3 toxins are internalized into the cytosol of immature human monocyte-derived DCs; Figure S2: C3 toxins are not internalized into the cytosol of human endothelial HeLa cells; Figure S3: Specific Internalization of eGFP (green)-labeled C3bot and C3bot_E174Q_ into early endosomes (red) of immature human monocyte-derived DCs ex vivo.
