# Supplementary material for: Clostridial C3 Toxins Enter and Intoxicate Human Dendritic Cells
Source: Toxins (Basel). 2020 Sep 1;12(9):563. doi: 10.3390/toxins12090563 (PMC7551598; doi:10.3390/toxins12090563)
Supplement: Supplementary file 1 [file toxins-12-00563-s001.pdf]

Supplementary Materials: Clostridial C3 Toxins Enter and Intoxicate Human Dendritic Cells

Maximilian Fellermann, Christina Huchler, Lea Fechter, Tobias Kolb, Fanny Wondany, Daniel Mayer, Jens Michaelis, Steffen Stenger, Kevin Mellert, Peter Möller, Thomas Barth, Stephan Fischer and Holger Barth

Supplementary Results

Clostridial C3 Toxins are Internalized into the Cytosol of Immature Human Monocyte-Derived DCs

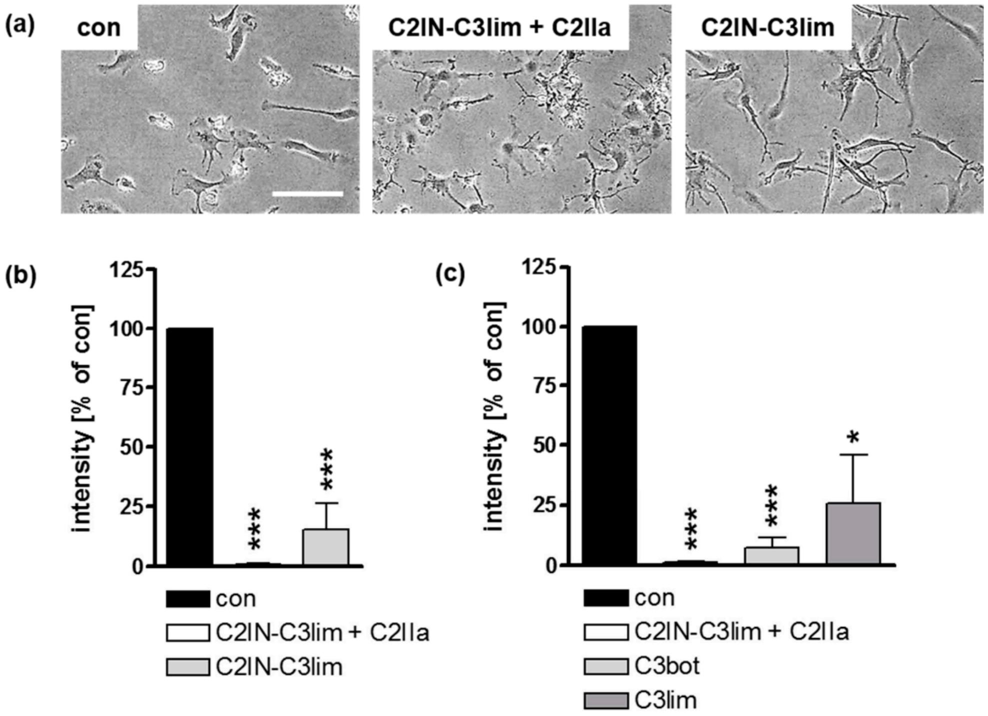

Figure S1: C3 toxins are internalized into the cytosol of immature human monocyte-derived DCs. (**a**) Immature DCs were treated at 37 °C with C2IN-C3lim/C2IIa (5/8.5 nM), C2IN-C3lim (80 nM), or were left untreated (con). Representative phase contrast images after 3.5 h are shown. Scale bar correspond to 50 µm and holds for all images. (**b**) Subsequently, cells were washed, lysed, and incubated with the respective freshly added C3 toxin (300 ng) and biotin-NAD+ (10 µM) for 30 min at 37 °C. Afterwards, cell lysates were transferred to SDS-PAGE and Western blot analysis to visualize biotin-labeled, i.e. ADP-ribosylated Rho proteins utilizing peroxidase-coupled streptavidin. Equal protein loading was confirmed via Ponceau S staining of the membrane. Densitometrical analyses from several experiments (normalized to Ponceau S loading control) are given as mean ± SD (n = 4). (**c**) Immature DCs were treated at 37 °C with C2IN-C3lim/C2IIa (5/8.5 nM), C3bot (160 nM), C3lim (160 nM) or were left untreated (con). After 3.5 h, detection and quantification of ADP-ribosylated Rho was performed like described above in (**b**). Values are given as mean ± SD (n = 3). (**b**,**c**). Significance was tested using a Student's t test (* $p < 0.05$, *** $p < 0.001$).

C3 Toxins do not Enter the Cytosol of HeLa Cells, Confirming the Cell-Type Selectivity

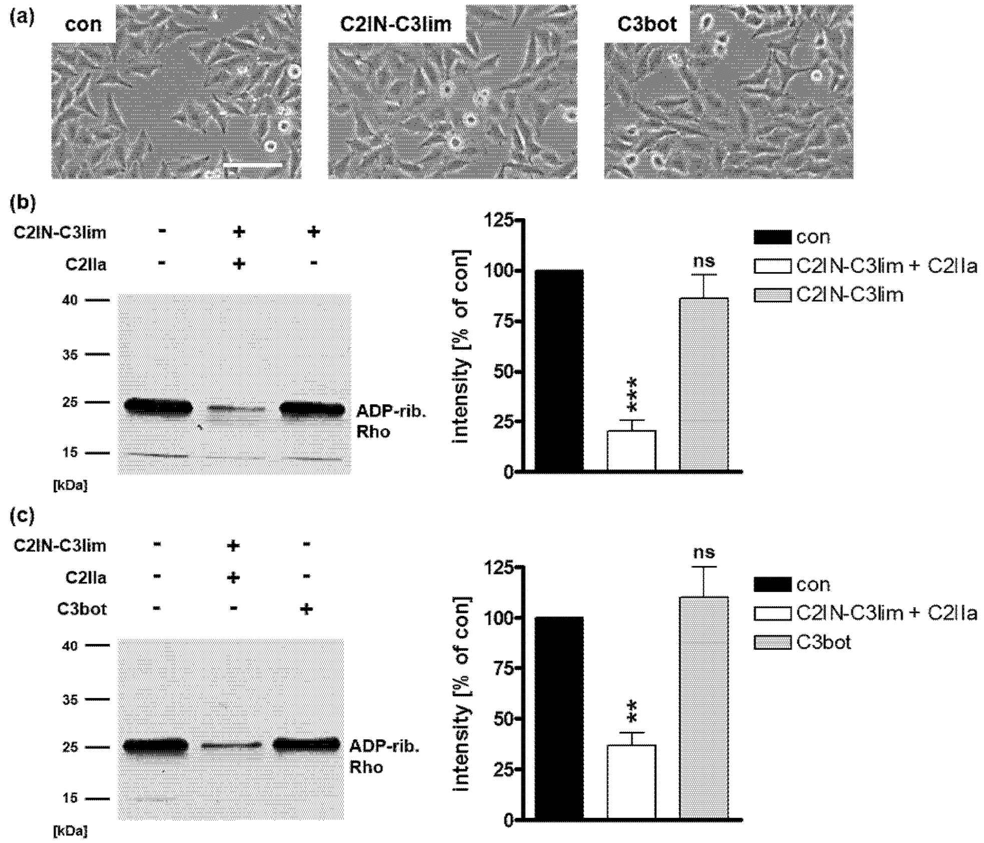

Figure S2: C3 toxins are not internalized into the cytosol of human endothelial HeLa cells. (**a**) HeLa cells were treated at 37 °C with C2IN-C3lim/C2IIa (20/66 nM), C2IN-C3lim (80 nM), C3bot (160 nM), or were left untreated (con). Representative phase contrast images taken after 5 h are shown. Scale bar corresponds to 50 µm and holds for all images. (**b**) Subsequently, untreated cells and cells treated with C2IN-C3lim/C2IIa or C2IN-C3lim were washed, lysed, and incubated with freshly added C2IN-C3lim toxin (300 ng) and biotin-NAD+ (10 µM) for 30 min at 37 °C. (**c**) In parallel, untreated cells and cells treated with either C2IN-C3lim/C2IIa or C3bot were washed, lysed, and incubated with freshly added C3bot toxin (300 ng) and biotin-NAD+ (10 µM) for 30 min at 37 °C. Afterwards, all cell lysates were transferred to SDS-PAGE and Western blot analysis to visualize biotin-labeled, i.e. ADP-ribosylated Rho proteins (~21 kDa) utilizing peroxidase-coupled streptavidin. Equal protein loading was confirmed via Ponceau S staining of the membrane. Densitometrical analyses from several experiments (normalized to Ponceau S loading control) are given as mean ± SD (n = 4) in (**b**) and as mean ± SD (n = 2) in (**c**). Significance was tested using a Student's t test (ns = not significant, ** $p < 0.01$, *** $p < 0.001$).

Specific Internalization of C3bot into Early Endosomes of Immature DCs

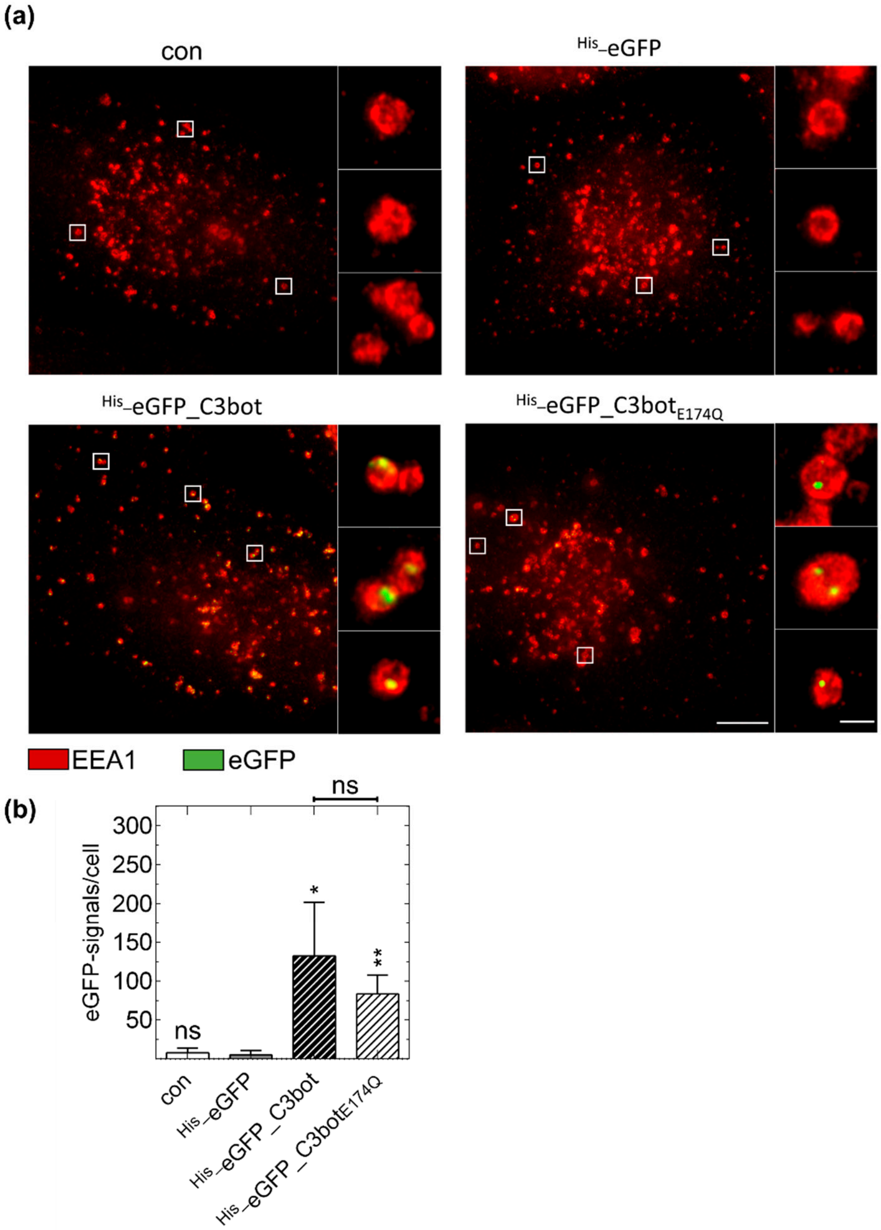

Figure S3: Specific Internalization of eGFP (green)-labeled C3bot and $C3bot_{E174Q}$ into early endosomes (red) of immature human monocyte-derived DCs ex vivo. (**a**) Isolated human monocytes were differentiated into immature DCs. DCs were treated with 250 nM ^{His}_eGFP_C3bot, ^{His}_eGFP_$C3bot_{E174Q}$, ^{His}_eGFP for 30 min at 37 °C, or left untreated (con). After subsequent immunostaining, STED super-resolution microscopic images were captured. Magnified areas of each image are marked with white squares. The experiment was repeated with DCs differentiated from monocytes of five individual and independent donors. Scale bar corresponds to 5 µm (0.5 µm for the magnifications) and holds for all images. (**b**) The detected green spots were quantified for each treatment (n = 5 donors). Significant differences compared to the ^{His}-eGFP samples were tested with Student's t test (ns = not significant, * $p < 0.05$, ** $p < 0.01$). Comparing the samples with ^{His}-eGFP_C3bot and ^{His}-eGFP_$C3bot_{E174Q}$ no significant differences were found as indicated above the graph.
